# HbA1c is a predictive factor of severe coronary stenosis and major adverse cardiovascular events in patients with both type 2 diabetes and coronary heart disease

**DOI:** 10.1186/s13098-023-01015-y

**Published:** 2023-03-20

**Authors:** Xiaojuan Jiao, Qin Zhang, Ping Peng, Yunfeng Shen

**Affiliations:** 1 Department of Endocrinology and Metabolism, The Second Affiliated Hospital of Nanchang University, No. 1, Minde Road, Donghu District, Nanchang, 330006 China; 2 Branch of National Clinical Research Center for Metabolic Diseases, Nanchang, 330006 China; 3 Institute for the Study of Endocrinology and Metabolism in Jiangxi Province, Nanchang, 330006 China

**Keywords:** Type 2 diabetes mellitus, Coronary heart disease, Glycated hemoglobin A1c, Coronary stenosis, Major adverse cardiovascular event

## Abstract

**Background:**

Coronary heart disease (CHD) is not only a macrovascular complication of type 2 diabetes mellitus (T2DM). Cardiovascular disease (CVD) is one of the leading causes of mortality among individuals with T2DM. Reducing the risk of adverse cardiovascular events (MACE) is crucial for the management of patients with CHD. This study aimed to investigate the effect of glycemic control on CHD severity and 3-point MACE (3p-MACE) risk in patients with T2DM and CHD.

**Methods:**

681 patients with both T2DM and CHD throughout October 2017 and October 2021 who were hospitalized in the second affiliated hospital of Nanchang university were included. A total of 300 patients were eventually enrolled in this retrospective cohort research. The severity of CHD in these patients was assessed, and the primary outcome during follow-up was recorded, with the primary result being the 3-point major adverse cardiovascular event (3p-MACE). The correlation between baseline glycated hemoglobin A1c (b-HbA1c) and the severity of CHD was evaluated by logistic regression analysis. The effect of b-HbA1c and follow-up HbA1c (f-HbA1c) levels on the risk of 3p-MACE were investigated by cox regression analysis.

**Results:**

b-HbA1c was positively correlated with the severity of CHD (r = 0.207, *p* = 0.001), and patients with b-HbA1c > 9% were more likely to have severe CHD. The HRs for b-HbA1c and f-HbA1c on the risk of 3p-MACE were 1.24 (95% CI 0.94–1.64, *p* = 0.123) and 1.32 (95% CI 1.02–1.72, *p* = 0.036), respectively. Patients with f-HbA1c   ≥8.6% had a higher risk of 3p-MACE than f-HbA1c < 8.6% (HR = 1.79, 95% CI 1.16–2.79, *p* = 0.009).

**Conclusion:**

In patients with both T2DM and CHD, b-HbA1c was an independent predictive factor of severe CHD. f-HbA1c was an independent predictive factor of 3p-MACE. Having the f-HbA1c below 8.6% significantly reduced the risk of 3p-MACE.

**Supplementary Information:**

The online version contains supplementary material available at 10.1186/s13098-023-01015-y.

## Background

Diabetes mellitus (DM) is a group of metabolic diseases characterized by hyperglycemia caused by inadequate or impaired insulin secretion or utilization. The global prevalence of DM is increasing at an alarming rate, with the number of adults with DM reaching 537 million (10.5%) worldwide in 2021, an increase of 74 million compared to 2019. The total number of people with DM is expected to increase to 643 million (11.3%) and 783 million (12.2%) worldwide by 2030 and 2045, respectively [1]. Type 2 diabetes mellitus (T2DM) accounts for around 90% of people with DM. Hyperglycaemia in T2DM can damage large vessels, and cardiovascular disease is the most common complication of T2DM with a high incidence [2]. Coronary Heart Disease (CHD) is a macrovascular complication in T2DM patients. It is manifested by atherosclerosis (AS) or (and) spasms of the coronary arteries, resulting in narrowing and occlusion of the cardiovascular lumen and inadequate or interrupted blood supply to the myocardium. Coronary angiography (CAG) is the gold standard for diagnosing CHD. Patients with both T2DM and CHD tend to have diffuse, occlusive lesions throughout the coronary arteries, with a high risk of bleeding, ulceration, and calcification. Approximately two-thirds of DM patients die from cardiovascular disease and stroke [3]. Reducing the risk of adverse cardiovascular events (MACE) is crucial for the management of patients with CHD.

Glycosylated hemoglobin A1c (HbA1c) is a marker of the average blood glucose level over the past 8–12 weeks. HbA1c has less variability, is unaffected by acute factors such as stress and exercise, and better represents relative long-term glycemic control [4]. With elevated HbA1c, the relative risk of complications in T2DM increases significantly, including neuropathy, retinopathy, and microangiopathy. Although studies have demonstrated that intensive glycemic control can reduce the occurrence of microangiopathy in T2DM patients, it remains controversial in reducing macrovascular complications and improving clinical outcomes. [5, 6]

This study aimed to investigate the effect of HbA1c at different times on CHD severity and MACE risk in patients with both T2DM and CHD and guide clinicians to appropriate glucose management in these patients.

## Methods

### Study population

Consecutive hospitalized T2DM patients diagnosed with CHD by CAG at the Second Affiliated Hospital of Nanchang University from October 2017 to October 2021 were included through the hospital medical database. CHD patients included stable angina, unstable angina, myocardial infarction (MI), and those who underwent emergent PCI. This study excluded patients with malignant tumors, renal failure requiring hemodialysis, previous diagnosis of CHD, or other heart diseases without pre-CAG HbA1c (b-HbA1c). Follow-up was performed after CAG, and patients lost to follow-up were excluded. For assessing the level of HbA1c at follow-up, subjects without at least three HbA1c measurements during follow-up were also excluded.

### Definitions

Diagnostic criteria for T2DM refer to WHO criteria [7]. Diagnostic criteria for CHD refer to ACC/AHA coronary angiography guidelines: The severity of coronary artery stenosis is assessed by CAG, and CHD is diagnosed when one or more of the coronary arteries have luminal stenosis of more than 50% [8].

Gensini score is a widely used angiographic scoring system for quantifying the severity of CHD [9]. The Gensini score was calculated by two clinicians independently, under the guidance of a cardiologist, following the Gensini score guidelines [10]. Step 1 is to calculate the severity score of the coronary lesion, step 2 is to apply a multiplication factor to each lesion score based upon the location of the lesion in the coronary tree, and step 3 is to sum the lesion severity scores. The detailed calculation of the Gensini score was shown in Additional file 1: Table S1. We were prespecified that the patients would be divided into three groups based on tertiles of the Gensini score (T1: < 29; T2: 29–72; T3: > 73) and defined these three groups as mild, moderate, and severe CHD. To assess the glycemic control of the patients, b-HbA1c was divided into < 6%, 6%–7%, 7%–8%, 8%–9%, and ≥ 9% groups according to the different levels.

### Baseline data collection

During hospitalization for CAG, baseline characteristics were collected. Demographic information such as age, sex, and body mass index (BMI) was written. Medical histories such as hypertension, stroke, and related medication were recorded. Laboratory tests such as HbA1c, fasting serum glucose (FSG), and fasting total cholesterol (TC) were analyzed. Coronary angiography results and type of coronary artery disease were consulted. HbA1c was measured by High-Performance Liquid Chromatography (HPLC). Some patients would be excluded if they have any condition shortening erythrocyte survival or decreasing mean erythrocyte age (e.g., recovery from acute blood loss, hemolytic anemia, chronic kidney disease).

### Follow-up outcomes and data

Follow-up was begun after patients underwent CAG and ended in February 2022. Patients were followed up via the electronic medical record system or telephone, and laboratory tests and outcome events during the follow-up period were recorded. The primary outcome was the 3-points MACE (3p-MACE), defined as cardiovascular death, nonfatal stroke, and nonfatal myocardial infarction. Cardiovascular death was defined as death attributable to an ischemic cardiovascular cause like fatal MI, stroke, or sudden death secondary to a presumed cardiovascular cause in this high‐risk population [11]. The definition of nonfatal stroke refers to ischemic and hemorrhagic strokes [12]. Nonfatal myocardial infarction is an acute myocardial infarction (AMI) that does not result in death. AMI is the presence of acute myocardial injury detected by abnormal cardiac biomarkers in the setting of evidence of acute myocardial ischemia [11]. Laboratory indicators for follow-up included HbA1c, TC, Triglyceride (TG), High-density lipoprotein cholesterol (HDL-C), Low-density lipoprotein cholesterol (LDL-C), and microalbuminuria (MAU).

### Statistical analyses

Continuous variables were expressed as the mean ± standard deviation, and categorical variables were expressed as frequencies and percentages. Baseline and follow-up demographic and clinical characteristics were compared with the Pearson χ2 test for categorical variables and analysis of variance for continuous variables. Correlations between two continuous variables were analyzed using Spearman's correlation analysis. The correlation between two ranked variables was assessed using Spearman's Rank Correlation.

Patients with mild and moderate CHD were categorized into non-severe CHD groups, thus dividing patients into severe and non-severe CHD groups. Binary logistic regression and ordered logistic regression analyses were used to assess the association of b-HbA1c as a continuous and categorical variable with severe CHD, respectively. To evaluate other risk factors of the severity of coronary artery disease, we included the baseline indicators in Table 1 in logistic regression one by one. The independent risk of HbA1c (b-HbA1c and f-HbA1c) and insulin therapy for 3p-MACE was assessed by age, sex, and history of hypertension in a multivariate cox proportional risk model. Interactions between f-HbA1c and insulin therapy on 3p-MACE outcomes were assessed in a cox model. For each patient, person-months of follow-up were counted from the date of diagnosis of CHD to the date of diagnosis of 3p-MACE or February 2022, whichever came first. Therefore, AMI and stroke leading to death were not counted in the 17 nonfatal strokes and 15 nonfatal infarction events. To analyze the effect of f-HbA1c on 3p-MACE, cumulative event incidence estimates were plotted according to f-HbA1c levels using the Kaplan–Meier technique. Differences between event-free curves were assessed with the log-rank test.

**Table 1:** Baseline demographic, clinical, and angiographic data of patients

N	Overall	No-event	3p-mace event	*P* value
	300	269	31	
Demographics				
Age, years	65.3 ± 10.2	65.2 ± 10.1	65.9 ± 11.1	0.708
Male	193 (64.3%)	176 (65.4%)	17 (54.8%)	0.244
BMI (kg/m^2^)	24.9 ± 2.4	24.9 ± 2.4	24.9 ± 2.5	0.975
Systolic BP, mmHg	133.6 ± 22.3	133.2 ± 22.6	137.5 ± 19.2	0.316
Diastolic BP, mmHg	77.7 ± 13.5	77.7 ± 13.6	78.2 ± 12.0	0.838
Heart Rate,	82.1 ± 16.4	81.8 ± 16.5	84.9 ± 15.5	0.314
Medical history				
Diabetes duration, years	9.8 ± 7.0	9.7 ± 7.0	10.9 ± 6.8	0.366
Hypertension	206 (68.7%)	180 (66.9%)	26 (83.9%)	0.054
Stroke	28 (9.3%)	24 (8.9%)	4 (12.9%)	0.471
ACEI/ARB	92 (30.7%)	80 (29.7%)	12 (38.7%)	0.305
Insulin	98 (32.7%)	84 (31.2%)	4 (45.2%)	0.117
Laboratory values				
HbA1c, (%)	8.0 ± 1.8	7.9 ± 1.7	8.7 ± 2.4	0.013
Fasting glucose, mmol/L	9.2 ± 4.2	9.1 ± 3.9	10.4 ± 5.8	0.095
TC, mmol/L	4.5 ± 1.2	4.5 ± 1.2	4.6 ± 1.2	0.881
TG, mmol/L	1.9 ± 1.2	1.8 ± 1.2	2.0 ± 1.4	0.514
HDL-C, mmol/L	1.1 ± 0.3	1.0 ± 0.3	1.1 ± 0.4	0.080
LDL-C, mmol/L	2.8 ± 0.9	2.8 ± 0.9	2.7 ± 0.8	0.629
eGFR, mL/min/1.73 m2	83.4 ± 38.8	84.2 ± 39.2	76.1 ± 35.1	0.268
BNP (pg/ml)	391.1 ± 680.8	395.1 ± 708.4	356.8 ± 366.6	0.767
LVEF, %	59.6 ± 13.9	59.7 ± 14.3	58.9 ± 9.7	0.762
Angiographic data				
Gensini score	55.6 ± 41.5	54.8 ± 41.5	62.5 ± 41.2	0.328
Myocardial infarction	102 (34.0%)	92 (34.2%)	10 (32.3%)	0.829
PCI	179 (59.7%)	160 (59.5%)	19 (61.3%)	0.846
Severity of CHD				0.580
Mild	92 (30.7%)	84 (31.2%)	8 (25.8%)	
Moderate	70 (23.3%)	64 (23.8%)	6 (19.4%)	
Sever	138 (46.0%)	121 (45.0%)	17 (54.8%)	

Values given as mean ± SD or number (percentage), ACEI: angiotensin-converting enzyme inhibitor, ARB: Angiotensin Receptor Blocker, TC: Total Cholesterol, TG: Triglyceride, HDL-C: High-Density Lipoprotein Cholesterol, LDL-C: Low-Density Lipoprotein Cholesterol, eGFR: estimated Glomerular Filtration Rate, LVEF: Left Ventricular Ejection Fraction, PCI: Percutaneous Coronary Intervention

Two-tailed* p* values < 0.05 were considered significant. SPSS software (IBM SPSS Statistics 23) and R software (R 3.6.1) were used for statistical analysis.

## Results

### Baseline characteristics and follow-up data of patients

**Fig. 1:**
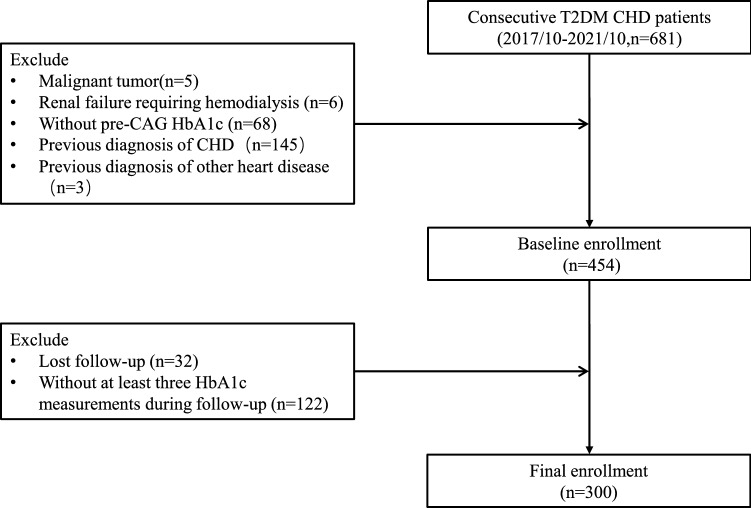
Flow chart of the recruitment procedure. T2DM: type 2 diabetes mellitus; CHD: coronary heart disease; CAG: coronary angiography

A total of 681 patients with both T2DM and CHD were included through the hospital medical database. Patients who had malignant tumors (n = 5), renal failure requiring hemodialysis (n = 6), previous diagnosis of CHD (n = 145), or other heart diseases (n = 3) without b-HbA1c (n = 68) were excluded. During the follow-up, 32 patients were lost to follow-up, and patients (n = 122) without at least three HbA1c measurements were excluded. Ultimately, 300 subjects were included in the study analysis. (Fig. 1) The mean age of the patients was 65 years, the duration of diabetes was approximately 10 years, the mean b-HbA1c was about 8.0%, 193 (64.3%) of them were male, 206 (68.7%) patients had hypertension, 138 (46.0%) patients had severe CHD, 102 (34.0%) patients had acute myocardial infarction (AMI), and 179 (59.7%) patients underwent percutaneous coronary intervention (PCI) (Table 1).

The mean follow-up period was 24 months, and 31 patients (10.3%) experienced 3p-MACE (11 cardiovascular death, 17 nonfatal strokes, and 15 nonfatal infarction). Compared with the event-free patient group, b-HbA1c levels (8.7% vs7.9%, *p* = 0.013) and f-HbA1c (8.6% vs 7.8%,* p* = 0.008) were significantly higher in the 3p-MACE group (Table 2).

**Table 2:** Follow-up Laboratory data of patients

N	Overall	No-event	3P-MACE	*P* value
	300	269	31	
Laboratory values				
f-HbA1c, (%)	7.9 ± 1.7	7.8 ± 1.6	8.6 ± 2.3	0.008
f-TC, mmol/L	3.9 ± 1.0	3.9 ± 1.0	4.0 ± 1.0	0.655
f-TG, mmol/L	1.6 ± 0.9	1.6 ± 0.9	1.7 ± 1.1	0.510
f-HDL-C, mmol/L	1.1 ± 0.3	1.1 ± 0.3	1.1 ± 0.3	0.394
f-LDL-C, mmol/L	2.9 ± 5.4	2.9 ± 5.6	2.8 ± 3.7	0.957
f-MAU, mg/L	133.2 ± 282.5	126.9 ± 247.3	187.5 ± 495.9	0.259

f: follow-up

### Distribution characteristics of b-HbA1c and CHD severity

**Fig. 2:**
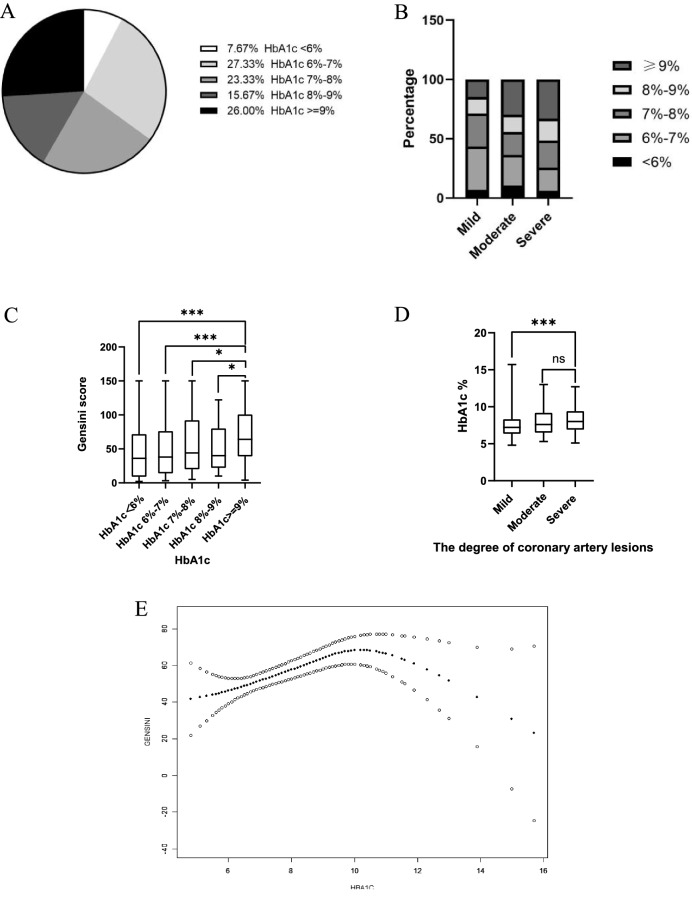
Distribution characteristics of b-HbA1c and severity of CHD. **A** The proportion of different levels of b-HbA1c in patients with both T2DM and CHD; **B** Percentage of b-HbA1c at different levels in patients with mild, moderate, and severe coronary stenosis, respectively; **C**: Differences in Gensini score values between different levels of b-HbA1c groups; **D**: Differences in b-HbA1c values between different grades of coronary stenosis groups; **E**: Smoothed curve fit for b-HbA1c and Gensini scores; *P < 0.05, **P < 0.01, ***P < 0.001, ns = no significance

Among these patients, 26.0% had a b-HbA1c ≥ 9%, next to those with b-HbA1c 6%–7% (27.3%) (Fig. 2A). The group with mild CHD had the highest proportion of patients with a b-HbA1c in the 6%–7% range (34.8%), whereas the group with severe CHD had the highest proportion of patients with a b-HbA1c in the ≥ 9% range (32.6%) (Fig. 2B). As b-HbA1c levels increased, Gensini scores tended to increase. With patients in the b-HbA1c < 6%, 6%–7%, 7%–8% and 8%–9% ranges all having significantly lower Gensini scores than those with b-HbA1c ≥ 9% (*p* < 0.05) (Fig. 2C). The severity of CHD increased gradually with b-HbA1c. Compared to the severe CHD group, patients in the mild CHD group had significantly lower b-HbA1c values (*p* < 0.001) (Fig. 2D).

The distribution characteristics of b-HbA1c and CHD severity in the patients revealed a strong correlation. By plotting the curve fit between b-HbA1c and the Gensini score (Fig. 2E), it could be seen that the two were roughly positively correlated. When b-HbA1c was used as a continuous variable, b-HbA1c was significantly associated with Gensini score values (r = 0.207, *p* = 0.001). When b-HbA1c was used as a categorical variable, b-HbA1c was significantly positive correlated with CHD severity (r_s_ = 0.180, *p* = 0.002). Additionally, duration of diabetes, fasting glucose, LDL-C, and BNP were risk factors for severe CHD, as shown in Additional file 1: Table S2.

### Baseline -HbA1c is an independent predictive factor of severe CHD

**Fig. 3:**
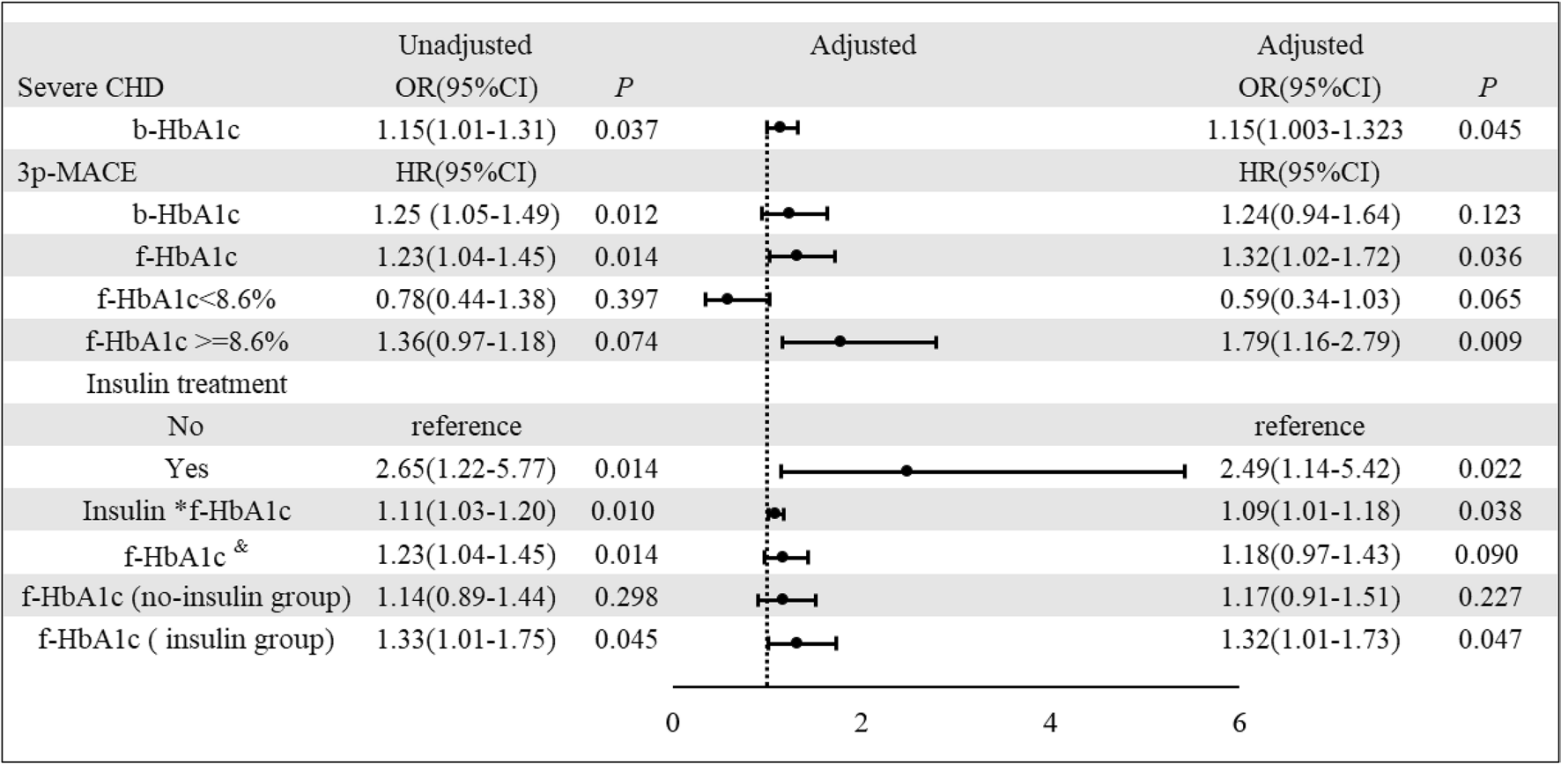
Forest plots of logistic regression analysis Odds ratios with 95% CI for severe stenosis and Forest plots of cox regression analysis hazard ratios with 95% CI for 3p-MACE.The following items were included as covariates in the multifactorial logistic regression analysis: demographic factors (age, sex, BMI), medical history (hypertension, stroke, diabetes duration), laboratory tests on admission (FBG, LDL-C, HDL-C, TG, CHOL, eGFR, BNP level) in the multifactorial logistic regression analysis. Age, sex and history of hypertension were adjusted in the multifactorial cox regression analysis. &: adjusted the interaction item Insulin *f-HbA1c

Univariate logistic regression showed that b-HbA1c was a risk factor for severe CHD in patients with T2DM (OR = 1.149, *p* = 0.037). Multivariate logistic regression further demonstrated that b-HbA1c was an independent risk factor for severe CHD in patients with T2DM (OR = 1.151, *p* = 0.046) (Fig. 3). The risk of severe CHD increased by approximately 15% for every 1% increase in b-HbA1c value. Therefore, b-HbA1c could be considered a predictor of having severe CHD.

### Baseline -HbA1c as a risk factor for major adverse cardiovascular events

Univariate cox regression analysis showed that b-HbA1c was a risk factor for the prognosis of 3p-MACE in T2DM patients with CHD (HR = 1.25, 95% CI 1.05–1.49, *p* = 0.012). However, it was not found to be an independent risk factor by multifactorial analysis (HR = 1.24, 95% CI 0.94–1.64, *p* = 0.123) (Fig. 3). A subgroup analysis of the study population was performed to identify factors influencing the association between b-HbA1c and the risk of prevalence of 3p-MACE. It showed that b-HbA1c was not a risk factor for the 3p-MACE in some subgroups (e.g., age ≥ 65 years, hypertension, duration of diabetes ≥ 12 months, insulin treatment, PCI, severe CHD, etc. *p* > 0.05) (Additional file 1: Figure S1.)

### Follow-up HbA1c is an independent predictive factor of major adverse cardiovascular events

**Fig. 4:**
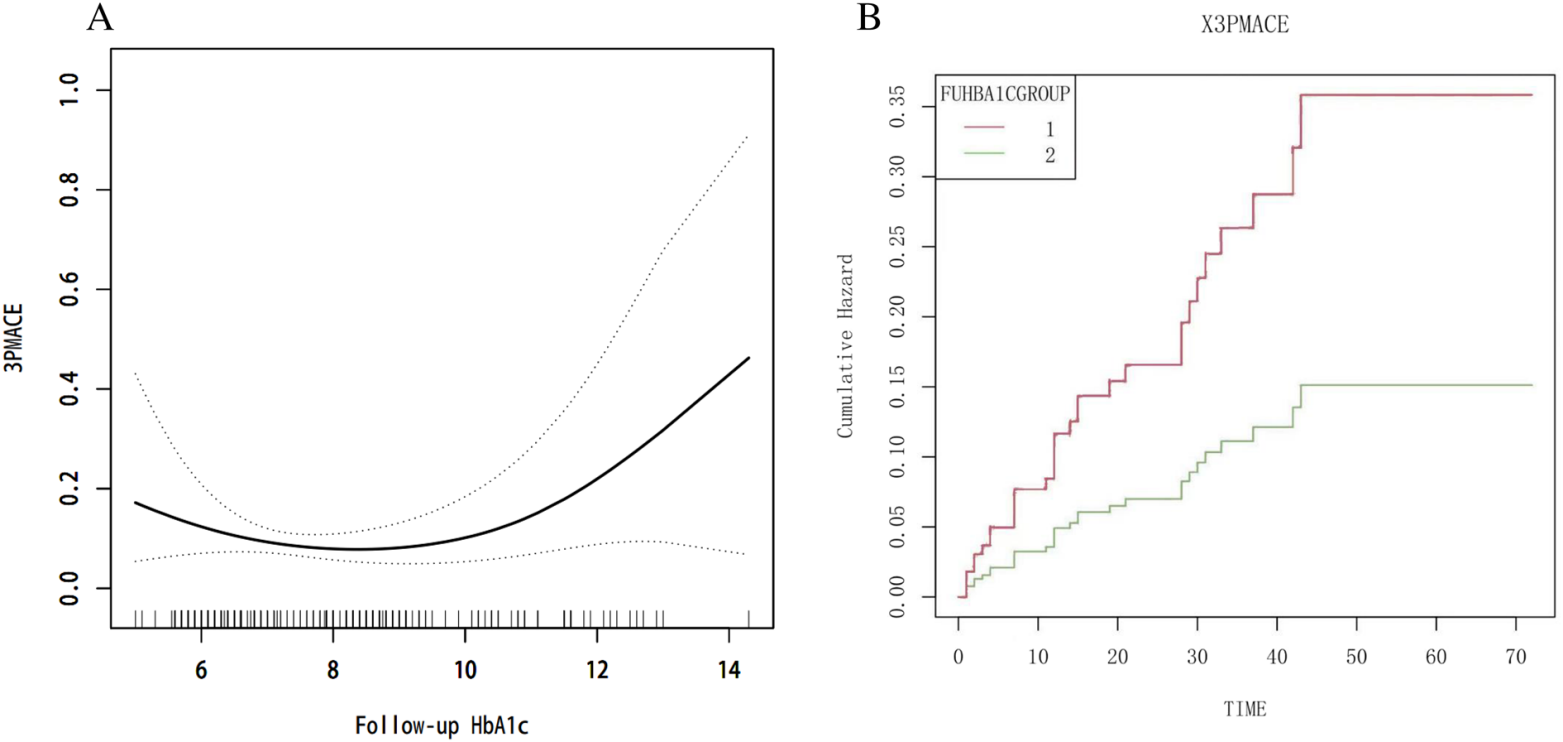
f-HbA1c and the risk of 3p-MACE. **A** Smoothed curve fit of f-HbA1c values and the risk of 3p-MACE; **B** Cumulative risk of 3p-MACE between groups with different f-HbA1c values. f-HbA1c GROUP 1 = f-HbA1c  ≥ 8.6%, f-HbA1c GROUP 2 = f-HbA1c  < 8.6%

By univariate (HR = 1.23, 95% CI 1.04–1.45, *p* = 0.014) and multivariate cox regression analysis (HR = 1.32, 95% CI 1.02–1.72, *p* = 0.036), f-HbA1c was found to be an independent risk factor for the 3p-MACE (Fig. 3). A curve fit of f-HbA1c to 3p-MACE risk was plotted (Fig. 4A), and a curve cut-off point of 8.6% was found using threshold effects analysis. For patients with f-HbA1c ≥ 8.6%, f-HbA1c was an independent risk factor for 3p-MACE (HR = 1.79, 95% CI 1.16–2.79, *p* = 0.009), whereas for patients with f-HbA1c < 8.6%, the association was not statistically significant (HR = 0.59, 95% CI 0.34–1.03, *p* = 0.065) (Fig. 3). The cumulative incidence of 3p-MACE between groups with different f-HbA1c values was plotted (Fig. 4B). It showed a significant difference in cumulative event rates between the two groups. Those patients with f-HbA1c < 8.6% had a significantly lower risk of 3p-MACE than those with f-HbA1c ≥ 8.6% (*p* = 0.014).

### Insulin treatment and prognosis of CHD patients

Interestingly, insulin treatment was strongly associated with the risk of 3p-MACE in these T2DM with CHD patients by cox regression analysis. Compared with non-insulin treatment, insulin treatment had a higher risk of 3p-MACE (HR = 2.49, 95% CI 1.14–5.42, *p* = 0.022) (Fig. 3). However, b-HbA1c and f-HbA1c were higher in the insulin-treated group than in the noninsulin-treated group (Table 3). Interactions between f-HbA1c and insulin therapy on 3p-MACE outcomes were assessed. The results show that the interaction was significant, and the P value for the interaction term was reported (*p* = 0.038). After including the interaction item of insulin*f-HbA1c in the adjusted cox model, the HR for f-HbA1c on the risk of 3p-MACE was 1.18 (95%CI 0.97–1.43, p = 0.090). The unadjusted HR was 1.23 (95%CI 1.04–1.45, p = 0.014). For patients with insulin treatment, f-HbA1c was an independent risk factor for 3p-MACE (HR = 1.32, 95% CI 1.01–1.73, *p* = 0.047), whereas for patients without insulin treatment, the association was not statistically significant (HR = 1.17, 95% CI 0.91–1.51,* p* = 0.227). (Fig. 3).

**Table 3:** HbA1c levels between insulin-treated and non-insulin-treated groups

	No Insulin treatment	Insulin treatment	Z	*P value*
b-HbA1c (%)	7.7 (6.5–8.8)	7.9 (6.8–9.6)	− 1.986	0.047
f-HbA1c (%)	7.4 (6.5–8.3)	7.88 (6.9–9.4)	− 2.083	0.005

*b-HbA1c* baseline HbA1c, *f-HbA1c* follow-up HbA1c

## Discussion

This study found that patients with T2DM with poor glycemic control had a significantly increased risk of severe coronary stenosis compared to those with good glycemic control. b-HbA1c was a risk factor but not an independent risk factor for the 3p-MACE in T2DM with CHD patients. In contrast, f-HbA1c was an independent risk factor for 3p-MACE in these patients. 3p-MACE in patients with both T2DM and CHD was significantly decreased when f-HbA1c < 8.6%.

T2DM promotes the development and progression of atherosclerosis, including not only traditional risk factors such as genetic factors, hyperglycemia, obesity, lipid metabolism disorders, sex hormone abnormalities, advanced age, and smoking but also nontraditional risk factors such as hyperinsulinemia, insulin resistance, diabetic hypercoagulable state, diabetic endothelial dysfunction, advanced glycosylation end products, oxidative stress, diabetic inflammation, microproteinuria, and hyperhomocysteinemia [13]. HbA1c is not only used to diagnose T2DM but can also be used to identify people at high cardiovascular risk. The higher the HbA1c level, the greater the risk of cardiovascular events in patients with T2DM [14].

Hyperglycaemia damages the cardiovascular system and induces atherosclerosis through several mechanisms, such as endothelial cell damage, oxidative stress, and imbalances in the coagulation and fibrinolytic systems, leading to diffuse coronary artery disease. Although previous studies have shown a positive correlation between high levels of HbA1c and the severity of coronary artery disease, these have been limited to specific types of coronary artery disease [14–16]. The risk of b-HbA1c levels to coronary artery disease in a population of patients not differentiated by CHD type is currently unknown. This study assessed the severity of coronary lesions based on CAG using the Gensini score and found a positive correlation between b-HbA1c levels and the severity of coronary lesions, in line with previous studies [17, 18].

The Gensini score is a scientific evaluation standard of coronary artery lesions, taking into account the number, location, and severity of stenosis of coronary artery lesions [10]. And it is a useful tool for assessing the severity of CHD [19]. Higher levels of b-HbA1c (HbA1c > 9%) were associated with more severe CHD when HbA1c was analyzed as a categorical variable, suggesting that elevated b-HbA1c predicts increased CHD severity.

Many studies have examined the prognostic impact of baseline blood glucose levels on patients with CHD. Still, most have been limited to patients with AMI or PCI, suggesting a positive association between b-HbA1c and poor prognosis [20–26]. b-HbA1c significantly predicted adverse cardiovascular events at prognosis [27]. A Chinese population-based study also showed no significant difference in prognosis between groups with different b-HbA1c levels in patients with T2DM combined with CHD who underwent PCI [28]. This study found that b-HbA1c was a risk factor for 3p-MACE in T2DM with CHD patients but was not an independent risk factor and was influenced by other factors such as age, duration of diabetes, hypertension, etc. Therefore, in patients with both T2DM and CHD, b-HbA1c alone cannot be used as an indicator to predict the long-term risk of 3p-MACE.

The prognosis of patients with CHD is closely related to glycemic control at follow-up. The poorer the glycemic control at follow-up and the need for insulin control, the higher the risk of developing 3p-MACE. It showed a higher incidence of 3p-MACE in the insulin-treated group. Meanwhile, there was relatively poorer glycemic control in the insulin-treated group. The DIGAMI-2 study in 2005 found that intensive glycemic control reduced mortality in heart attack patients [29]. The subsequent DIGAMI-1 in 2014 found that glycemic control by insulin therapy significantly reduced 1-year mortality in patients with acute infarction compared to the conventional treatment group. This finding contradicts the results of this study [30]. We found the risk of 3p-MACE in patients with both T2DM and CHD increased with the higher f-HbA1c in the insulin treatment group. In comparison, it was not significant in the patients without insulin treatment.

Furthermore, 3p-MACE increased with higher glycemia when f-HbA1c ≥ 8.6%. The results of this study were consistent with the 2020 Chinese guidelines [31], which state that the recommendation of HbA1c < 8.0% for T2DM patients with a long duration of diabetes, a history of cardiovascular disease, or a very high risk of cardiovascular disease is consistent. And patients with f-HbA1c values above the national 8.0% threshold present an increased risk of 3p-MACE.

The novelty of our study was that in addition to measuring the pre-CAG HbA1c as the level of the b-HbA1c value, the post-CAG HbA1c was also tested as the f-HbA1c. Thus, it reflects not only the relationship between glycemic control before CAG and CHD severity but also the impact of different stages of glycemic control on the prognosis of patients with both T2DM and CHD. Second, further threshold effect analyses were performed to determine which range of HbA1c was more likely to lead to severe CHD and adverse cardiovascular events and to guide clinicians in developing an individualized glycaemic management strategy for patients when b-HbA1c and f-HbA1c levels were found to correlate with the severity of coronary stenosis and the risk of 3p-MACE.

However, there were some limitations in this study. As this was a retrospective cohort study, much of the data were obtained from hospital databases or by reviewing patients' medical records, and some of the data were missing. For example, there were no specific descriptions of smoking and drinking history, data on whether patients had been treated with statins before hospitalization were incomplete, and many patients did not have a urine albumin creatinine ratio, so these indicators were not included in the data analysis. Smoking [32], alcohol history [33], statin treatment [34], and urine microalbumin [35] have been shown to be strongly associated with cardiovascular disease, so the absence of such data at baseline may have had some impact on the results. As study excluded patients on tumor and renal failure dialysis at the time of inclusion, which significantly reduced the number of deaths of patients due to these causes, and only three deaths from other causes were observed at follow-up. So the cox regression model did not include death from other causes as a competing risk, which may have affected the study results. Furthermore, as the assessment of coronary angiograms could only be based on what was available in the medical records, it was not possible to score angiogram images according to the latest 2019 Gensini scoring criteria [36] or to use other scoring metrics, such as the SYNTAX score [37], to assess the severity of CHD. The use of a different scoring system may affect the results.

## Conclusions

In conclusion, b-HbA1c was positively associated with the severity of CHD and was a risk factor for adverse cardiovascular events in T2DM with CHD patients, but not an independent risk factor. Whereas f-HbA1c was an independent risk factor. Hence b-HbA1c was an independent predictive factor of severe CHD, and f-HbA1c was an independent predictive factor of 3p-MACE. Patients with f-HbA1c above 8.6 were at the highest risk for 3p-MACE.

## Supplementary Information

**Additional file 1: Table S1.** Step-by-step algorithm for the Gensini Score calculation. **Table S2.** Other risk factors of severe CHD. **Figure S1.** A subgroup analysis of the relationship between baseline HbA1c and risk of 3p-MACE.

## Data Availability

The data of this study may be available on reasonable request to the corresponding author.

## Abbreviations

DM: Diabetes mellitus
T2DM: Type 2 diabetes mellitus
CHD: Coronary heart disease
AS: Atherosclerosis
HbA1c: Glycated hemoglobin A1c
b-HbA1c: Baseline HbA1c
f-HbA1c: Follow-up HbA1c
3p-MACE: 3-Point major adverse cardiovascular event
CAG: Coronary angiography
BMI: Body mass index
FSG: Fasting serum glucose
TC: Total cholesterol
TG: Triglyceride
HDL-C: High-density lipoprotein cholesterol
LDL-C: Low-density lipoprotein cholesterol
eGFR: Estimated glomerular filtration rate
LVEF: Left ventricular ejection fraction
MAU: Microscale albuminuria
AMI: Acute myocardial infarction
PCI: Percutaneous coronary intervention
ACEI: Angiotensin-converting enzyme inhibitor
ARB: Angiotensin receptor blocker
