# Supplementary material for: HbA1c is a predictive factor of severe coronary stenosis and major adverse cardiovascular events in patients with both type 2 diabetes and coronary heart disease
Source: Diabetol Metab Syndr. 2023 Mar 20;15:50. doi: 10.1186/s13098-023-01015-y (PMC10026512; doi:10.1186/s13098-023-01015-y)

Table S1. Step-by-step algorithm for the Gensini Score calculation.

| Step 1 Calculation of the severity score for coronary lesions | |
| --- | --- |
| Degree of stenosis (%) | Severity Score |
| <25 | 1 |
| 25-50 | 2 |
| 51-75 | 4 |
| 76-90 | 8 |
| 91-99 | 16 |
| 100 | 32 |
| Step 2 A multiplying factor is applied to each lesion score based upon its location in the coronary tree | |
| Segment | multiplying factor |
| Left Main Artery (LM) | 5 |
| Left circumflex artery (LC) | |
| pLC | 2.5 |
| dLC | 1 |
| Posterior Lateral (PL) | 0.5 |
| Posterior descending artery (R-PDA) | 1 |
| Anterior descending artery (LAD) | |
| pLAD | 2.5 |
| mLAD | 1.5 |
| Diagonal branches D1 | 1 |
| Diagonal branches D2 | 0.5 |
| Right Coronary Artery (RCA) | 1 |
| Step 3 Sum of the lesion severity scores | |

Table S2. Other risk factors of the severe CHD

|  | OR (95%CI) | *P* |
| --- | --- | --- |
| Diabetes duration | 1.034(1.001, 1.069) | 0.046 |
| Fasting glucose | 1.077(1.018, 1.140) | 0.010 |
| LDL-C | 1.274(1.095, 1.632) | 0.045 |
| BNP | 1.001(1.000, 1.001) | 0.014 |

Figure S1. A subgroup analysis of the relationship between baseline HbA1c and risk of 3p-MACE.


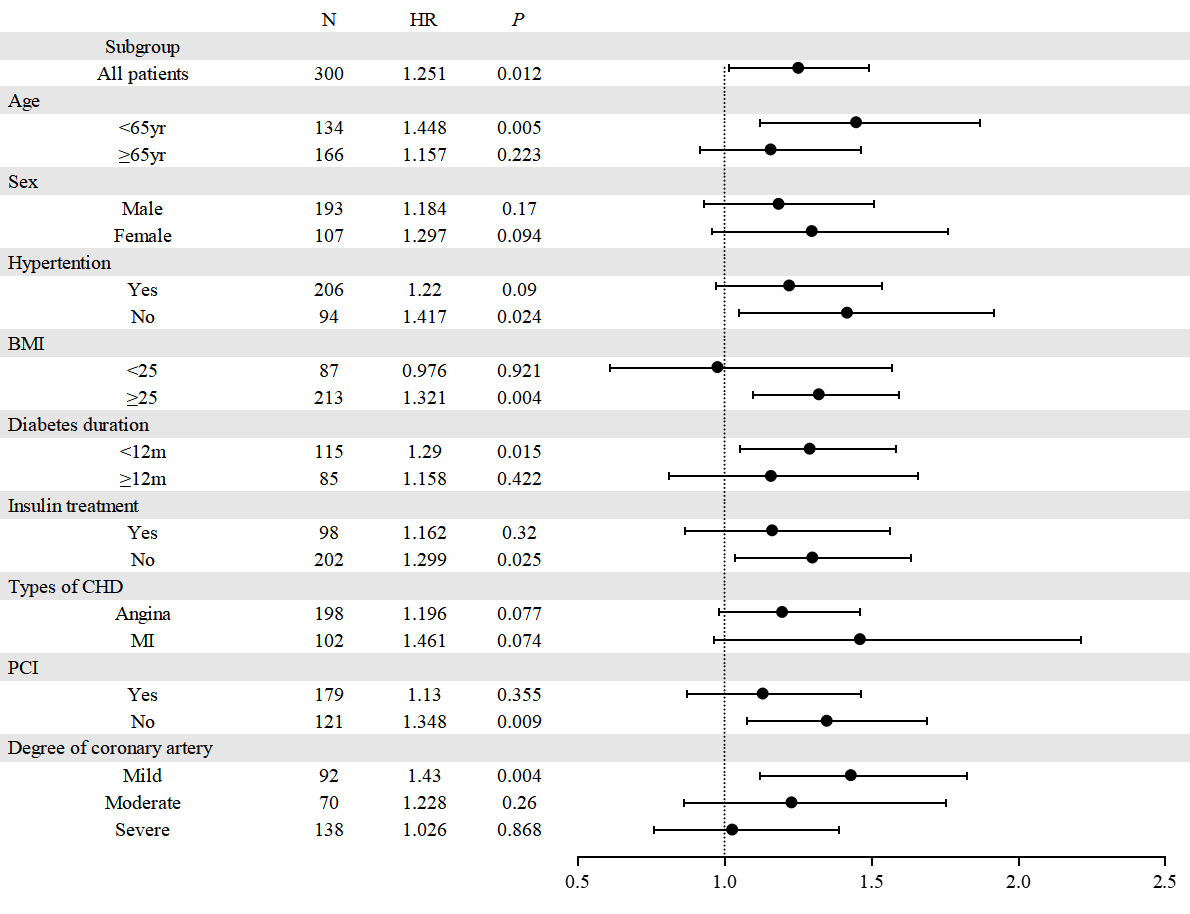

Supplement: Supplementary file 1 — Additional file 1: Table S1. Step-by-step algorithm for the Gensini Score calculation. Table S2. Other risk factors of severe CHD. Figure S1. A subgroup analysis of the relationship between baseline HbA1c and risk of 3p-MACE. [file 13098_2023_1015_MOESM1_ESM.docx]
